# Mesenchymal Stem Cells in Soft Tissue Regenerative Medicine: A Comprehensive Review

**DOI:** 10.3390/medicina59081449

**Published:** 2023-08-10

**Authors:** Ayesha Rehman, Aditya Nigam, Luigi Laino, Diana Russo, Celestino Todisco, Giuseppe Esposito, Fabiano Svolacchia, Federica Giuzio, Vincenzo Desiderio, Giuseppe Ferraro

**Affiliations:** 1 Department of Experimental Medicine, University of Campania “Luigi Vanvitelli”, Via L. Armanni 5, 80138 Naples, Italy; ayesha.rehman@unicampania.it (A.R.); aditya.nigam@unicampania.it (A.N.); 2 Multidisciplinary Department of Medicine for Surgery and Orthodontics, University of Campania “Luigi Vanvitelli”, Via L. Armanni 5, 80138 Naples, Italy; luigi.laino@unicampania.it (L.L.); dianarusso96@gmail.com (D.R.); giuseppe.ferraro@unicampania.it (G.F.); 3 ASL Napoli 3 Sud., 80059 Torre del Greco, Italy; c.todisco@aslnapoli3sud.it (C.T.); g.esposito@aslnapoli3sud.it (G.E.); 4 Departments of Medical-Surgical Sciences and Biotechnologies, Sapienza University of Rome, 00118 Rome, Italy; fabiano.svolacchia@gmail.com; 5 Department of Sciences, University of Basilicata, Via Nazario Sauro 85, 85100 Potenza, Italy; federica.giuzio@unibas.it; 6 U.O.S.D. of Plastic Surgery A.O.R “San Carlo”, 85100 Potenza, Italy

**Keywords:** mesenchymal stem cells, regenerative medicine, tissue regeneration, tissue engineering

## Abstract

Soft tissue regeneration holds significant promise for addressing various clinical challenges, ranging from craniofacial and oral tissue defects to blood vessels, muscle, and fibrous tissue regeneration. Mesenchymal stem cells (MSCs) have emerged as a promising tool in regenerative medicine due to their unique characteristics and potential to differentiate into multiple cell lineages. This comprehensive review explores the role of MSCs in different aspects of soft tissue regeneration, including their application in craniofacial and oral soft tissue regeneration, nerve regeneration, blood vessel regeneration, muscle regeneration, and fibrous tissue regeneration. By examining the latest research findings and clinical advancements, this article aims to provide insights into the current state of MSC-based therapies in soft tissue regenerative medicine.

## 1. Introduction

The repair and restoration of different tissues throughout the human body is a complex and dynamic process, crucial for maintaining the body’s overall integrity and function in response to various injuries and disease states [1,2]. Soft tissues, including skin, muscles, blood vessels, nerves, and fibrous tissues, play diverse roles and contribute to the body’s integrity and functionality [3]. These tissues are involved in essential functions such as temperature regulation, locomotion, oxygen and nutrient transport, sensory perception, and structural support.

Traditional therapeutic approaches for soft tissue injuries or degenerative disorders have relied on conservative therapies such as physical therapy, medication, and surgery. While these methods offer some benefits like comfort and tissue healing, they often have limitations [4]. Severe soft tissue injuries may surpass the body’s innate regenerative capacity, leading to inadequate repair. Scar tissue formation during healing can impede proper tissue functionality and slow recovery. Furthermore, conventional treatments may struggle to restore tissue structure and function, especially in complex soft tissue environments with diverse cell types and intricate tissue architectures [5].

In recent years, regenerative medicine has emerged as a promising field aiming to overcome the limitations of traditional treatments and provide innovative approaches for soft tissue repair and regeneration [5]. Regenerative medicine is a broad term that encompasses a range of strategies, such as tissue engineering, cellular therapy, gene therapy, and the use of biomaterials, to restore damaged or diseased tissues to their original form and function [6,7,8]. Out of cell-based therapies, mesenchymal stem cells (MSCs) have garnered significant attention due to their unique characteristics and regenerative potential. MSCs possess self-renewal and differentiation capabilities, including the ability of MSCs to differentiate into cell types specific to soft tissues. This ability makes MSCs a particularly promising avenue for soft tissue regeneration [9]. Researchers and clinicians have been exploring the potential of MSCs in various types of soft tissue regeneration, including craniofacial and oral soft tissues, blood vessels, muscles, and fibrous tissues.

## 2. Mesenchymal Stem Cells

MSCs are a type of multipotent stem cell derived from various sources, such as bone marrow (BM-MSCs), adipose tissue (AD-MSCs), umbilical cord (UC-MSCs), and dental pulp (DP-MSCs) [10]. These cells have gained attention in regenerative medicine due to their ability to self-renew, modulate the immune response, and differentiate into multiple cell lineages. Their differentiation potential into fibroblasts, chondrocytes, osteoblasts, and adipocytes makes them particularly attractive for tissue repair and regeneration [11].

**Figure 1:**
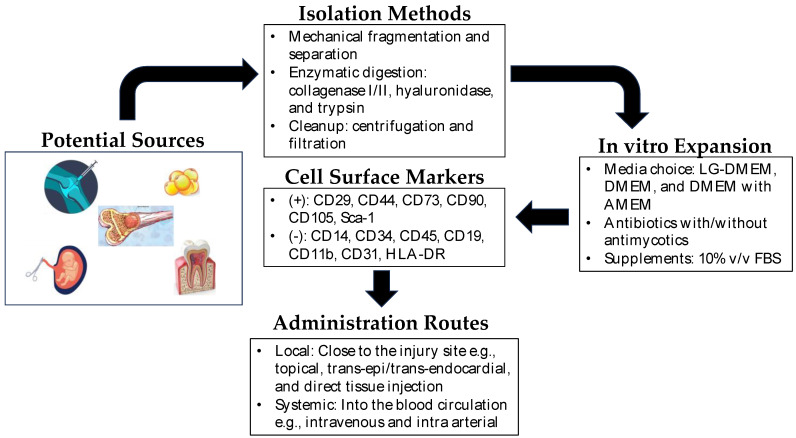
Mesenchymal stem cells in regenerative medicine. Depicted above is a brief overview of the isolation, expansion, and administration of mesenchymal stem cells in regenerative therapy. Potential sources of MSCs include synovium, bone marrow, dental pulp, amniotic fluid, umbilical cord, and adipose tissue. The isolation of MSCs from these sources is very straightforward, and MSCs are easily expandable in vitro. In clinical settings, they can be administered locally near the site of the injured tissue or directly into the blood (intravenous/intra-arterial) as systemic administration.

The extraction methods for MSCs vary depending on the tissue source. For example, BM-MSCs are typically harvested from bone marrow aspiration, followed by isolation and expansion in culture. AD-MSCs are obtained through liposuction or surgical excision of adipose tissue, which is then processed to extract the MSCs. UC-MSCs are derived from umbilical cord tissue or blood, while DP-MSCs are obtained from dental pulp extracted for therapeutic or orthodontic purposes [12]. Each source has its advantage and disadvantage as cell yield, proliferation capacity, and differentiation potential, which should be considered based on specific applications. Figure 1 shows the general scheme for the isolation and expansion of MSCs for clinical applications. 

Characterizing MSCs is essential to confirm their identity and quality. The International Society for Cellular Therapy (ISCT) has established minimal criteria to define MSCs, including adherence to plastic, expression of specific surface markers (CD73, CD90, CD105), and absence of hematopoietic markers (CD45, CD34, CD14, or CD11b, CD79a, or CD19, HLA-DR) [13]. Table 1 details the list of notable cell surface markers essential for the isolation and characterization of different MSCs. Additionally, MSCs can be evaluated for their multilineage differentiation potential, typically by inducing differentiation into adipogenic, osteogenic, and chondrogenic lineages and analyzing lineage-specific markers and matrix production [14]. Table 1 also summarizes the prominent differentiation of various MSCs isolated from different sources, in particular, lineages for therapeutic applications. 

MSCs have already received approval from the U.S. Food and Drug Administration (FDA) for some clinical applications. For instance, BM-MSCs are used to treat complications arising from hematopoietic stem cell transplantation, such as graft-versus-host disease (GVHD) [36]. BM-MSCs have immunomodulatory properties, suppressing the immune response and promoting tissue repair. Promising results from clinical trials led to FDA approval of MSC-based therapies, and ongoing investigations explore their potential in cartilage and bone repair, wound healing, and autoimmune disorders [9]. 

### 2.1. MSCs in Craniofacial and Oral Soft Tissue Regeneration

Craniofacial and oral soft tissue regeneration aims to restore damaged or lost tissues in the head and face region resulting from trauma, congenital conditions, surgical interventions, or cancer resections [37]. This anatomically complex area includes craniofacial districts such as the maxillofacial region, oral cavity, mandibular area, and facial bones. Injuries to these tissues can have significant functional and aesthetic consequences, impacting speech, chewing, oral health, and facial aesthetics. 

Recent studies have explored the potential of MSCs in craniofacial and oral soft tissue regeneration. For instance, Chen et al. (2017) studied calcium phosphate scaffold and endothelial cell co-culture with MSCs from various sources for their respective angiogenic and osteogenic characteristics in vivo and demonstrated that these constructs are promising for craniofacial bone reconstruction application [38]. Srinivasan et al. (2021) demonstrated the in vitro bone regenerative capacity of MSCs from human neural crest stem cells in comparison to bone marrow-derived MSCs for craniofacial bone reconstruction [39]. The embryonic origin of MSCs was related to the greater regenerative capacity as it resulted in enhanced mineralization and differential expression of genes linked to FGF in a 3D polycaprolactone-tricalcium phosphate (PCL-TCP) scaffold system. 

Researchers have investigated that the combination of MSC-derived exosomes with scaffolds promotes tissue repair and regeneration in craniofacial applications without cell transplantation [40]. The study concludes that the exosomes from dental pulp-derived mesenchymal stem cells (DP-MSCs) cause osteogenic differentiation and mineralization of bone marrow stromal cells when released in a controlled manner from a biodegradable scaffold and facilitate craniofacial bone reconstruction. Similarly, Liu et al. (2021) demonstrated, in a cell-free approach, that exosomes generated from MSCs had a superior effect on angiogenesis, effectively promoting craniofacial soft tissue regeneration [41]. These data provide a novel strategy to use MSCs in regenerative medicine of oral tissue repair. Hydrogels have also received remarkable attention due to their capacity for stimulating bone reconstruction in craniofacial regeneration [42]. Chu et al. (2021) also utilized moldable gelatin-nanohydroxyapatite cryogel with allogeneic BM-MSCs for craniofacial bone regeneration [43]. 

#### 2.1.1. Maxillofacial and Mandibular Bone Reconstruction

One area of research focuses on the reconstruction of maxillofacial and mandibular bone defects using MSCs [44,45]. Yin et al. (2019) studied the application of human amniotic mesenchymal cells (AM-MSCs) in maxillary sinus floor elevation rabbit models and observed better implant osseointegration and bone regeneration that can be useful to optimize dental implantation in the future [46]. Sha et al. (2019) demonstrated the effective reconstruction of complex maxillofacial defects with the application of a novel three-dimensional (3D) porous uncalcined and unsintered hydroxyapatite/poly-d/l-lactide (3D-HA/PDLLA) composite loaded with human MSCs in a pilot animal study [47]. Stutz et al. (2021) studied BM-MSCs added on bone morphogenic protein 2 (BMP)-functionalized polycaprolactone biomembranes implanted in a maxillary bone lesion that resulted in acceleration of the bone remodeling process and hence a promising technology for future clinal usage [48]. Similarly, many in vivo studies have demonstrated significant evidence for the application of MSCs for the regeneration and repair of mandibular defects. For instance, Wang et al. (2019) conducted a study using MSCs derived from human adipose tissue in combination with a porous nano-HA/collagen/PLA scaffold. They successfully regenerated bone tissue and improved functional outcomes in a preclinical model [49]. Similarly, Probst et al. (2020) showed the bone regeneration capacity of AD-MSCs seeded into tri-calcium phosphate-poly(D, L-lactide-co-glycolide) scaffolds in minipigs with large mandibular defects [50]. Furthermore, Song et al. (2020) reported that the supplementation of Cystathionine γ-lyase-H^2^S facilitates the healing process and the osteogenic differentiation of human mandibular bone marrow mesenchymal stem cells (HM-BMMSCs) in mice models of a mandibular defect [51]. 

Moreover, a clinical trial by Gjerde et al. (2018) demonstrated that MSCs can enhance bone formation and improve functional outcomes in patients with significant mandibular defects [52]. The study included 11 subjects with severe mandibular ridge resorption that were treated with BM-MSCs using biphasic calcium phosphate granules as a scaffold. Following 4 to 6 months of the healing period, the bone volume was adequate for dental implantation. These findings highlight the potential of MSC-based approaches for restoring mandibular bone defects.

#### 2.1.2. Dental Pulp Regeneration

In the last few years, the pulp regeneration of non-vital teeth has received ever-increasing attention. For instance, the application of dental-derived MSCs for pulp regeneration has gained popularity with the development of validated animal models and clinical trials. Since ample blood supply is vital for pulp regeneration, vascular regeneration of the graft has become a prerequisite for the beforementioned. In a recent study, rod-like DP-MSC-made sheets were used for transplantation into the root canals that further differentiated into odontoblast-like mineralizing cells and pulp-like tissues. These differentiated tissues had better access to oxygen and nutrients and the ability to inhibit cell apoptosis and turned out to be very similar to the natural dental pulp [53]. Another study also reported the use of vascularized DP-MSC construct for dental pulp regeneration [54]. The outer cellular layer of the construct expressed makers for endothelial differentiation at a higher level than the inner cellular layer, leading to the formation of a vascular-like structure. Transplantation of this construct in a non-vital human tooth implanted inside an immunodeficient mouse demonstrated that the construct facilitated a better blood supply and enhanced regenerative capability. Hence, dental pulp regeneration with self-constructed biomimetic material is of interest due to its relation to angiogenesis for regenerative dental medicine. In this context, Li et al. (2023) introduced the use of transglutaminase-crosslinked gelatins as a scaffold for seeding DP-MSCs and showed that it increases cell migration, stimulates odontogenic differentiation of DP-MSCs, and promotes the formation of dentin [55].

Interestingly, Yuan et al. (2022) indicated the use of a less invasive and faster source of stem cells in contrast to DP-MSCs, which otherwise involved the time-taking process of tooth extraction. In vivo analysis of stem cells from human-exfoliated deciduous teeth loaded in injectable simvastatin-functionalized gelatin methacrylate cryogel microspheres demonstrated vascularized pulp-like tissue regeneration [56]. 

Apart from DP-MSCs, stem cells derived from apical papilla (SCAP) dental follicle stem cells (DFSCs) have also been explored for their regenerative capabilities. 

For instance, Liu et al. (2021) demonstrated the application of SCAP cultured with hypoxia-mimicking cobalt-doped multi-walled carbon nanotube nanocomposites in enhancing the angiogenic potential for dental tissue engineering [57]. Meng et al. (2022) studied N-acetylcysteine-treated human DFSCs that have MSC-like properties and potential for regeneration of the alveolar bone [58]. The study concludes that N-acetylcysteine treatment enhances DFSC’s ability for osteogenesis and repairs alveolar bone via ROS scavenging. 

#### 2.1.3. Periodontal Regeneration

Periodontitis involves the destruction of periodontal support tissues, including alveolar bone resorption, attachment loss, and matrix destruction [59]. For the reconstruction of oral tissue, MSCs have shown promise in promoting the formation of periodontal ligament-like structures, crucial for tooth support and stability [60]. Hu et al. (2019) demonstrated the transplantation of DP-MSCs and DP-MSC sheets directly onto the periodontal defects in pigs to study the effect of DP-MSCs on the treatment of periodontal regeneration [61]. The study found that both DP-MSCs and DP-MSC sheet groups showed bone regeneration, but the bone mass in the DP-MSC sheet group was higher linked with the greater efficiency of sheet-like DP-MSCs. Previously, Cao et al. (2015) also observed a higher percentage of bone mass while studying DP-MSC sheets and DP-MSCs transfected with hepatocyte growth factor (HGF) in the context of periodontal alveolar bone regeneration [62]. In vivo studies demonstrated successful regeneration of periodontium, dentin, and pulp-like tissues after the implantation of the sandwich formation of human DP-MSC sheet/human-treated dentin matrix/matrigel. Several studies have also revealed the role of the extracellular matrix (ECM) derived from pulp tissues as a reliable source for site-specific tissue regeneration. Alqahtani et al. (2018) showed that transplantation of the ECM derived from decellularized pulp tissues in swine led to neovascularization and the formation of dental pulp-like tissue. Further, due to the differentiation of DP-MSCs into cementoblasts, mineralized tissues are constructed into the root canal. However, it is generally thought that inhibitors of cementogenesis are required to avoid mineralization during pulp regeneration in the root canal [63]. Cui et al. (2022) investigated the regenerative potential of mineralized ECM/dental pulp stem cells microspheroids (MMCMs) in oral tissue engineering, specifically in promoting periodontal tissue regeneration. Their research demonstrated the formation of functional periodontal ligament-like structures, offering a promising avenue for treating severe periodontitis [64]. Periostin, a potent biomolecule for periodontitis homeostasis, is shown to be highly expressed by DFSCs and plays an important role in DFSCs’ osteogenic abilities [65]. DFSCs have been validated to stimulate periodontal regeneration in an inflammatory microenvironment through the regulation of macrophages via human recombinant periostin loaded into the collagen matrix [66].

#### 2.1.4. MSCs for Anti-Aging

Moreover, MSC-based therapies have been also explored for regenerating facial soft tissues, such as skin and adipose tissues in terms of anti-aging therapy. Aging leads to the loss of collagen fibers from the skin, and elastic fibers become disorganized [67]. In aged skin, there is an increased oxidant activity and an increased extracellular matrix degradation caused by increased production of matrix metalloproteases [68]. Skin rejuvenation therapies focused on the prevention and reversal of skin aging are in high demand in our societies. Adipose-derived MSCs have proved to be a reliable starting material for skin rejuvenation therapy because of their efficient re-epithelialization capability and ability to secrete certain growth factors necessary for skin regeneration [69]. In a study, AD-MSCs were used in patients to promote facial skin rejuvenation [70]. Recently, Charles et al. (2015) injected expanded AD-MSCs (collected from fat removed by liposuction) into the aged facial skin of patients and observed promising changes in the histoarchitecture of the aged skin. There was an increase in the elastic fibers in the superficial layer of the dermis, and collagen and reticular fibers became more arranged [71]. In mice models of skin aging, AD-MSCs were capable of complete regeneration of solar elastosis [72]. Apart from directly injecting AD-MSCs into the facial tissue, a cell-free method of treatment is also feasible. In mice models of photo-aging, subcutaneous application of AD-MSC-derived extracellular vesicles leads to a reduction in skin wrinkles and initiation of epidermal cell proliferation. With the extracellular vesicle application, macrophage infiltration and reactive oxygen species generation were also reduced in this photo-aged mice model [72]. 

In addition to AD-MSCs, MSCs from other sources like AM-MSCs, BM-MSCs, and umbilical cord blood (UCB-MSCs) have also been used for craniofacial and oral soft tissue regeneration. Prakoeswa et al. (2019) showed that treatment with an AM-MSC-conditioned medium improved the clinical photo-aging symptoms like pore, wrinkle, and spot polarization in the treatment group [73]. UCB-MSCs are known for their rapid proliferation and immune-modulatory capabilities, and these MSCs are also easy to isolate. Kim et al. (2018) showed the presence of various growth factors, including epidermal growth factor (EGF), basic fibroblast growth factor (bFGF), transforming growth factor-beta (TGF-β), and several other factors, like HGF, collagen type 1, and growth differentiation factor (GDF) 11 in the cell culture media, when administered to human subjects, leading to wrinkle reduction in the treatment group [74]. These findings suggest that MSC-based approaches hold the potential for addressing cosmetic concerns and improving facial contouring.

#### 2.1.5. Wound Regeneration

Additionally, studies have investigated the use of MSCs in regenerating diabetic wounds. For example, Kato et al. (2017) evaluated the efficacy of AD-MSC sheets for repairing cranial injuries in diabetic rat models. Their findings indicated that allogenic transplantation of AD-MSC sheets in combination with artificial skin is applicable not only to diabetic wounds, but also to other intractable ulcers or burns, offering a potential therapeutic strategy for these disorders [75]. Furthermore, the transplant of induced pluripotent stem cell-derived MSCs (iMSCs) promoted thyroid cartilage regeneration in vivo, and some of the iMSCs differentiated into chondrogenic lineage cells [76]. These and other studies highlight the versatility and potential of MSCs in craniofacial and oral soft tissue regeneration. Combining MSCs with suitable scaffolds, growth factors, and tissue engineering strategies provides a promising approach for restoring form and function in patients with craniofacial and oral soft tissue injuries.

### 2.2. MSCs in Nerve Regeneration

MSCs have shown great potential in promoting nerve regeneration through various mechanisms, making them a promising therapeutic approach. MSCs can differentiate into astrocytes, neurons, and Schwann cell-like cells to support neural regeneration [77,78,79]. In addition to their differentiation capacity, MSCs secrete a variety of neurotrophic factors and growth factors, including brain-derived neurotrophic factor (BDNF), neural growth factor (NGF), and glia cell line-derived neurotropic factor (GDNF), which supports neuron survival and axonal regeneration [80]. The paracrine effects of MSCs on the local microenvironment contribute to immunomodulation, promotion of cell survival, and reduction in inflammation, creating a favorable milieu for nerve regeneration [81,82]. For instance, Chen et al. (2019) demonstrated the superiority of a human MSC-conditioned medium (CM) supplemented with bFGF, EGF, and patient plasma, namely, a neural regeneration laboratory medium (NRLM), on spinal cord injury in both in vitro and in vivo models [83]. The study showed that NRLM-conditioned media were better than standard media in inflammation reduction and neurite regeneration effects in vitro and improved functional restoration in spinal cord injury rats in vivo. Song et al. (2020) also demonstrated the modulatory effects of an MSC-CM and HGF in the presence of bone morphogenetic protein (BMP) 4, with or without a c-Met antibody, on neuronal stem cell differentiation and recovery of spinal cord injury [84]. Recently, Chouaib et al. (2023) also showed that a dental pulp-derived (DP)-MSC-CM can significantly stimulate neurite outgrowth in primary sensory neurons [85]. Chen et al. (2022) employed 3D-printed collagen/silk fibroin scaffolds to carry the secretome derived from UM-MSCs and demonstrated amelioration of neurological dysfunction after implantation of the scaffold in a rat model of spinal cord injury [86]. BM-MSCs and AD-MSCs are currently the most studied cell sources for CNS repair and hold similar neuronal differentiation capacities, but the number of cytokines and growth factors that AD-MSCs produced compared to BM-MSCs was shown to be significantly higher [87,88]. Apart from BM- and AD-MSCs, DP-MSCs have also shown regenerative capacity for nerve repair. Saez et al. (2019) investigated the therapeutic effects of MSCs derived from human dental pulp in a rat model of facial nerve injury. The MSCs promoted nerve regeneration and functional recovery by improving axonal regrowth and modulating the inflammatory response [89]. In short, the interaction between MSCs and nerve stem cells has unfolded a promising area of research in the field of neuronal regeneration [90]. Cui et al. (2022) studied the exosomes derived from UM-MSCs and showed that these exosomes inhibited the activation of microglia and astrocytes during brain injury, thereby promoting functional recovery in rats after traumatic brain injury [91]. Similarly, Li et al. (2022) studied exosomes from lipopolysaccharide-preconditioned BM-MSCs that were able to shift the pro-inflammation macrophage into a pro-regeneration macrophage and hence accelerated peripheral nerve regeneration via M2 macrophage polarization [92]. Recent studies have focused attention on the role of MSC-derived extracellular vesicles (EVs) for neuronal reconstruction [93,94]. Moreover, Zhou et al. (2023) summarized several in vivo experiments on gene editing that can effectively improve the repair effect of MSCs on nerve injury and confirms the effectiveness of the gene modification strategy [95]. Recent review papers have provided in-depth insight into the clinical application of MSCs for nerve regeneration in neurological disorders including Alzheimer’s disease (AD), Parkinson’s disease (PD), Huntington’s disease (HD), amyotrophic lateral sclerosis (ALS), multiple sclerosis (MS), and multiple system atrophy (MSA) [96,97,98]. These in vitro and in vivo studies collectively demonstrate the regenerative potential of MSCs in nerve tissues and highlight their ability to promote axonal regeneration, myelination, and functional recovery. Further research is warranted to optimize the delivery methods, dosage, and timing of MSC-based therapies to maximize their regenerative potential in clinical settings.

### 2.3. MSCs in Blood Vessel Regeneration

Several researchers have investigated the effects of MSCs on angiogenesis and vasculogenesis, the formation of new blood vessels. It is well known that MSCs play a role in vascularization. In vivo studies have demonstrated that MSCs function similarly to perivascular cells [99,100,101,102]. Furthermore, they can differentiate into endothelial cells and form capillary tube-like structures [100,101]. Recently, Jang et al. (2023) described that MSCs cultured with an endothelial cell culture medium and supplemented with vascular endothelial growth factor (VEGF) contribute to vasculogenesis by their sprouting capability in response to bFGF. These MSCs increase the angiogenic sprouting of human umbilical vein endothelial cells by secretion of a paracrine factor called HGF [103]. Many studies have focused on innovative approaches to enhance the therapeutic potential of MSCs in blood vessel regeneration. Genetic modification of MSCs to overexpress growth factors such as VEGF has shown promising results in several preclinical models [104,105,106]. This approach aims to enhance the pro-angiogenic properties of MSCs and promote more robust blood vessel formation.

The ability of MSCs to enhance angiogenesis has significant implications for restoring blood flow and tissue function. In addition to their involvement in blood vessel regeneration, MSCs exert immunomodulatory effects. These effects can indirectly influence the regeneration of blood vessels by regulating the inflammatory response and facilitating tissue repair [107,108]. Nammian et al. (2021) explored the application of allogenic BM-MSCs and AD-MSCs and found that they secrete immunomodulatory cytokines that are pro-angiogenic and lead to the formation of blood vessels [109]. Modulating the immune response is crucial to creating a favorable environment for blood vessel regeneration. MSCs represent a preference for making autologous tissue-engineered vascular grafts, as summarized by Afra et al. (2020) [110]. Apart from allogenic MSCs, autologous injections of AD-MSCs have also been shown to promote blood vessel regeneration [111]. Furthermore, Zhou et al. (2022) described that Pluronic F-127-encapsulated human UC-MSCs can restore the morphology of thin endometrium in a rat model with increased endometrium thickness and gland number and stimulated neovascularization. Moreover, IL-1β-primed human UC-MSC-derived conditioned media enhanced the concentration of vital angiogenesis factors bFGF, VEGF, and HGH [112]. Another report also explained that UC-MSCs migrate to the injured endometrium and promote the recovery of the thin endometrium and its structure and function [113]. Advancements in tissue engineering have paved the way for the development of MSC-seeded scaffolds for blood vessel regeneration. Combining MSCs with 3D-printed scaffolds has shown encouraging results in promoting the formation of functional blood vessels and improving tissue perfusion [114]. In this context, a menstrual blood mesenchymal stem cell (MB-MSC)-loaded collagen scaffold also induced endometrium regeneration in a rat model [115]. Tian et al. (2022) highlighted the research on tissue engineering of blood vessels using AD-MSCs as seed cells and self-assembled nano-polypeptide hydrogel for 3D printing of biological tissues [116]. These things considered, other studies have shown that MSCs can promote blood vessel formation and improve tissue perfusion in conditions such as myocardial infarction (MI) and hind limb ischemia [117,118,119]. In a study, MSCs were investigated in a murine model of hind limb ischemia. The researchers found that intramuscular injection of MSCs led to improved blood flow and increased capillary density in the ischemic limb, indicating their potential in promoting blood vessel regeneration [120]. Research showed how MSC transplantation resulted in increased blood vessel density and improved cardiac function in a rat model of myocardial infarction, suggesting the regenerative potential of MSCs in repairing damaged heart tissue [110,121,122,123].

MSCs have also been used in promoting blood vessel regeneration in a rabbit model of corneal neovascularization. The study demonstrated that topical application of MSCs led to reduced neovascularization and improved corneal transparency, indicating the neoangiogenic effects of MSCs [124], as also indicated by Khan et al. (2017) [125].

Other in vivo studies investigated the role of MSCs in promoting blood vessel regeneration in a diabetic foot ulcer model in rats. The researchers found that MSC transplantation accelerated wound healing, improved blood flow, and increased angiogenesis, highlighting their potential in the treatment of diabetic vascular complications [126,127]. Moreover, several reports have described the utility of extracellular vesicles derived from MSCs to promote angiogenesis in ischemia, wound healing, and myocardial infarction for tissue regeneration [128,129,130].

Menezes et al. (2020) studied human AD-MSCs for spinal cord injury in the rat model and found that human AD-MSCs, or their conditioned media, elicited angiogenesis by contributing to blood vessel maturation [131].

Collectively, these studies underscore the immense potential of MSCs in blood vessel regeneration. Their abilities to enhance angiogenesis, modulate the immune response, and synergize with tissue engineering approaches make MSCs a valuable asset in the quest for effective therapies in the field of vascular regeneration.

### 2.4. MSCs in Muscle Regeneration

Muscle tissue regeneration plays a crucial role in restoring the structure and function of injured or degenerated muscles. Skeletal muscle is a tissue that in homeostatic conditions performs regeneration activity with the help of tissue-resident muscle stem cells called satellite cells. These cells play a central role in skeletal muscle regeneration after injury. But, in diseases like Duchenne muscular dystrophy (DMD), these satellite cells accumulate abnormalities and are no longer capable of promoting cell proliferation and tissue regeneration [132]. MSCs have emerged as a promising approach for muscle regeneration due to their myogenic differentiation capacity and paracrine effects. MSCs can differentiate into myocytes and contribute to muscle fiber repair and regeneration, facilitating the restoration of muscle architecture and functionality. Additionally, MSCs secrete a range of growth factors, cytokines, and extracellular matrix components that promote myogenesis and angiogenesis while modulating the local inflammatory response, creating a favorable microenvironment for muscle regeneration and contributing to tissue repair [133,134,135].

In vitro studies have provided valuable insights into the potential of MSCs for muscle regeneration. Many authors investigated the myogenic differentiation capacity of human BM-MSCs and found that these cells expressed myogenic markers and fused with existing myotubes, indicating their ability to participate in muscle regeneration [136]. Similarly, Nitahara-Kasahara et al. (2021) demonstrated that DP-MSCs in animal models of Duchenne muscular dystrophy improved skeletal muscle dysfunction, providing further evidence of the myogenic potential of MSCs [137].

In vivo studies have also demonstrated the effectiveness of MSC-based approaches in muscle regeneration. AD-MSC transplantation in skeletal muscle not only differentiated into muscle-like cells but also secreted factors such as insulin-like growth factor-1 (IGF-1) and VEGF, promoting myogenesis and angiogenesis [138]. 

Efforts have also been made to check for the effectiveness of just the secretome of MSCs in skeletal muscle regeneration. In a study conducted by Robert Mitchell et al. (2019), the regenerative potential of a cell-free MSC secretome was demonstrated in vivo using a CTX mouse model of acute muscle injury [139]. The whole secretome of an AD-MSC consists of soluble proteins and extracellular vesicles (EVs) containing miRNA and soluble proteins as their cargo. The researchers showed that the AD-MSC secretome is capable of promoting cell proliferation and migration in vivo in the CTX mouse model for muscle injury. Then, they tried to asses the differential capability of secretome and EVs in muscle regeneration, and it was seen that both these fractions increased the cross-sectional area of newly regenerated muscle fibers and reduced the infiltration of macrophages with the EV fraction producing stronger effects. The EV fraction, in addition to stimulating cell proliferation and migration, also reduced the inflammation levels in the muscle injury and regeneration model, as supported by other studies [125,140]. Furthermore, several studies observed that MSCs improved muscle regeneration in volumetric muscle loss (VML), increased angiogenesis, and enhanced functional recovery [141]. These findings further support the beneficial effects of MSCs in muscle regeneration.

In addition to skeletal muscle regeneration, MSCs have shown promise in cardiac muscle regeneration. Studies have demonstrated that MSCs can improve myocardial function, attenuate adverse remodeling, and enhance angiogenesis in the context of myocardial infarction. For instance, Khan et al. (2017) investigated the effects of BM-MSC transplantation in a rat model of myocardial infarction and observed significant improvements in cardiac function, reduction in scar size, and increased angiogenesis [125].

A lot of research has been conducted to elucidate the therapeutic potential of MSC-derived exosomes in myocardial injury treatment. In the acute MI injury mouse model, it was shown that MSC-derived exosomes promote myocardial repair by reducing the inflammatory processes happening in the injured tissue. This immunosuppressive effect of MSC exosomes and MSCs is mediated through various routes, including immune tolerance reagents, like programmed death ligand 1, galectin 1, and membrane-bound TGF-β [142]; immuno modulatory miRNAs, like miR-24 and miR-29. MSC-Exo-delivered miR-182 slows down myocardial ischemia and reperfusion injury by modulating macrophage polarization [143].

Engineered mesenchymal stem cells, like MSCs overexpressing migration inhibitory factor [144], HGF [145], and stromal-derived factor 1 [146], have also been used in myocardial tissue repair and have shown promising results in myocardial tissue repair and regeneration, inhibiting myocardial cell apoptosis and promoting cardiac microvascular regeneration. Similarly, Chen et al. (2021) explored the therapeutic potential of UC-MSCs in cardiac regeneration and reported enhanced cardiomyocyte survival, neovascularization, and functional recovery [140]. These studies highlight the potential of MSCs, both through differentiation into cardiomyocyte-like cells and their paracrine effects, in promoting cardiac muscle regeneration.

The use of MSCs in muscle regeneration holds great promise for addressing various muscle-related conditions, including traumatic injuries, muscle wasting diseases, and myocardial infarction [147,148,149,150]. The combination of their myogenic differentiation capacity, secretion of growth factors, and modulation of the local microenvironment provides a multifaceted approach to promoting muscle tissue repair and functional recovery.

### 2.5. MSCs in Fibrous Tissue Regeneration

Fibrous tissues, such as tendons and ligaments, are susceptible to injury and often have limited regenerative capacity. MSCs have shown promise in fibrous tissue regeneration due to their ability to differentiate into tenocytes or fibroblasts and their paracrine effects that promote tissue healing and remodeling. MSCs can contribute to the regeneration of fibrous tissues by directly differentiating into tenocytes or fibroblasts, thereby enhancing the synthesis of extracellular matrix components and restoring tissue structure and function. Additionally, MSCs secrete a range of growth factors and cytokines that modulate the inflammatory response, promote angiogenesis, and stimulate endogenous repair mechanisms. 

In vitro studies have provided valuable insights into the potential of MSCs for fibrous tissue regeneration. For example, Yang et al. (2013) conducted in vitro experiments using AD-MSCs and demonstrated their ability to differentiate, in the presence of a tendon ECM, into fibroblast-like cells and promote the secretion of collagen and other extracellular matrix components, indicating their potential in ligament regeneration [151]. Similarly, through an indirect co-culture technique, Hwang et al. (2023) demonstrated tendon ECM-stimulated tenogenesis of BM-MSCs without the need for exogenous growth hormones or alterations to cellular viability [152]. In this context, Wang et al. (2020) found that the potent pro-tenogenic biological activity of tendon ECM is driven by integrin/ transforming growth factor-beta (TGF-β) signaling crosstalk [153]. Moreover, TGF-β2-activated AKT signaling contributes to the tenogenic differentiation of MSCs in the face of disruption of Smad3 signaling [154]. Another study discovered that BM-MSC-derived exosomes stimulate the activation of fibroblasts via PTEN and PI3K/Akt signaling pathways, which may serve as potential targets to further promote tendon–bone healing [155].

In contrast, studies have also used different pro-tenogenic factors to induce tenogenesis. An in vitro study has shown that equine AD-MSCs differentiate into tenocytes in response to the combination of platelet-derived growth factor-BB (PDGF-BB) and GDF-6 [156]. Another in vitro study has shown the promising role of GDF-7 for the regeneration of the tendon–bone interface due to its ability to differentiate into multiple lineages [157]. Li et al. (2023) found that a serum-free culture medium with a growth factor cocktail containing FGF-2, TGF-β3, and insulin-like growth factor-1 (IGF-1) with tendon-like substrate topography induced tenogenic differentiation of human BM-MSCs [158]. Furthermore, Zulkifli et al. (2023) discussed the role of hypoxia conditioning of MSCs to increase tenogenesis capacity, particularly focusing on the central role of hypoxia-inducible factor-1-alpha (HIF-1α) and its interaction with FGF-2 and TGF-β1 in regulating the differentiation of MSCs into tenocytes [159].

Researchers have also focused on tissue engineering approaches for the differentiation and reconstruction of fibrous tissues. Zhau et al. (2019) explored the potential of MSC sheets and bFGF loaded into knitted poly(lactide-co-glycolide)-fibrin gel scaffolds for damaged tendon reconstruction. They observed better reparative efficacy and tenogenesis of MSCs in situ model [160]. Xue et al. (2022) demonstrated that MSCs seeded on a mechanically strong and bioactive nanofibrous scaffold exhibit increased growth, proliferation, and tenogenic expression for tendon differentiation [161]. Berntsen et al. (2022) studied the potential of composite cell sheets fabricated from AD-MSCs for the engineering of the tendon–bone interface and showed favorable results for tissue differentiation [162]. Park et al. (2022) showed that mechanoactivation of aligned dense collagen seeded with human MSCs holds tenogenic differentiation and regeneration properties even in the absence of serum and growth factors, which can be translated into clinical applications in the future [163]. These studies highlight the recent research in 3D tissue engineering for fibrous tissue regeneration. 

In vivo studies have also demonstrated the effectiveness of MSC-based approaches in fibrous tissue regeneration. For instance, several studies explored the therapeutic potential of BM-MSCs and AD-MSCs in models of Achilles tendon injury and observed improved tendon structure, increased collagen organization, and enhanced mechanical properties [164,165,166]. Furthermore, many authors investigated the effects of MSC transplantation in animal models of medial collateral ligament (MCL) injury and reported accelerated MCL healing, improved tissue morphology, and enhanced biomechanical properties [167]. These in vivo findings further support the potential of MSCs in promoting fibrous tissue regeneration.

Moreover, studies have also investigated the use of MSC-based strategies in other fibrous tissues, such as the intervertebral disc and meniscus. For example, Khalid et al. (2023) evaluated the regenerative potential of human UC-MSCs plus Sox9 and TGF-β1 in an animal model of intervertebral disc degeneration and observed improved disc height, enhanced extracellular matrix synthesis, and reduced inflammatory response [168]. Similarly, other researchers investigated the effects of MSC transplantation in a model of meniscus injury and found increased meniscal cell density, improved meniscal structure, and enhanced meniscal extracellular matrix components [169]. 

Yea et al. (2023) compared BM-MSCs, UCB-MSCs, and UC-MSCs not only in a tensioned three-dimensional construct but also in a full-thickness tendon defect rat model and found that UC-MSCs are superior to other MSCs in their differentiation into tendon-like lineage cells and constitute a well-ordered tendon-like matrix in vitro. Moreover, UC-MSCs also have better regenerative properties in vivo in comparison to BM- and UCB-MSCs [170]. 

In short, these studies highlight the potential of MSCs in promoting fibrous tissue regeneration beyond tendons and ligaments.

The use of MSCs in fibrous tissue regeneration holds great promise for addressing various fibrous tissue-related conditions, including tendon and ligament injuries, degenerative disc disease, and meniscus damage. The combination of their differentiation capacity, secretion of growth factors, and modulation of the local microenvironment provides a multifaceted approach to promoting tissue repair and functional recovery in fibrous tissues.

## 3. Conclusions and Future Prospects

In conclusion, MSCs hold tremendous promise for tissue regeneration and repair due to their unique properties, including their ease of isolation and in vitro maintenance, multipotency, immunomodulatory effect, and paracrine activity. MSCs have shown great potential in various fields of regenerative medicine, as discussed in the article, such as oral and craniofacial tissue reconstruction, neuronal regeneration, as well as blood vessels, muscles, and fibrous tissue regeneration. The application strategies are numerous such as supplementation of growth factors, delivery with biodegradable scaffolds, and utilization of MSC-derived soluble molecules.

MSCs provide several benefits, but there are still a lot of obstacles to overcome. Understanding how the tissue environment affects the destiny and capabilities of MSCs is crucial in terms of lineage differentiation. The clinical transformation of MSCs is surely hampered by the difficult-to-unify differentiation potential, surface markers, and transcription of various tissue-derived MSCs. The distinct immunomodulatory characteristics of MSCs are vital for their roles, although it is yet unclear how the immune system in the context of MSCs is regulated. Moreover, to develop effective and secure regenerative medicine applications, it is also crucial to comprehend the paracrine route involved in the healing process controlled by MSCs.

Moreover, MSC-based tissue engineering provides a potential therapeutic approach in the field of regenerative medicine, notwithstanding the current difficulties. For MSC therapy, it is also crucial to enhance the cultural environment of MSCs and choose the proper scaffolds and induction factors. 

Overall, MSC-based approaches have demonstrated promising outcomes in tissue regeneration, offering a multifaceted approach through their differentiation capacity and secretion of growth factors. However, further research is still required to optimize MSC-based therapies, including standardizing protocols, addressing safety concerns, and understanding the mechanisms underlying their regenerative potential. With continued advancements in stem cell research and regenerative medicine, MSCs have the potential to revolutionize the treatment of various tissue-related conditions, bringing new hope for patients in need of effective and innovative therapies.

## Figures and Tables

**Table 1:** Profile of MSCs.

MSC Source Type	Surface Markers	Secreted Growth Factor Profile	Preferred Cell Type upon Differentiation	References
Adipose Tissue-derived	CD105, CD166, CD44, CD29, CD73, CD71	VEGF, HGF, BDNF, IGF, bFGF, TGF-β1	Adipocytes, chondrocytes, osteoblasts, cardiomyocytes, skeletal muscle cells, neurons, hepatocytes, and tenocytes	[15,16,17]
Dental Pulp-derived	CD105, CD29, CD44, CD146, CD271, CD34, CD117 CD90	VEGF, Ang-2, TGFα, EPO, SCF, FGF, PDGF-BB	Odontoblasts, osteoblasts, endothelial cells, and nerve cells	[18,19]
Synovial Fluid-derived	CD40, CD44, CD73, CD90, CD105, CD166	TGF-β, FGF	Chondrocytes, adipocytes, and osteocytes	[20,21,22]
Salivary Gland-derived	CD13, CD44, CD29, CD90	FGF, EGF	Hepatocytes and pancreatic beta cells	[23,24]
Bone Marrow-derived	CD105, CD90, CD73, CD44, CD166, CD29, CD146, CD271	G-CSF, GM-CSF, HGF, EGF, VEGF, FGF-2, FGF-4	Osteoblasts, adipocytes, and chondrocytes	[25,26,27,28]
Amniotic Fluid-derived	CD90, CD45, CD34, CD14, CD133, SSEA-4, CD117	TNF-α, TGF-β1, TNF-β, IFN-γ	Chondrocytes, osteocytes, adipocytes, neural cells, hepatocytes, alveolar epithelial cells, insulin-producing cells, cardiomyocytes, and germ cells	[29,30,31,32]
Umbilical Cord-derived	CD29, CD44, CD73, CD90, CD105, CD166	KGF, HGF, EGF, TNF-α, HGF, IGF1	Osteoblasts, chondrocytes, and adipocytes	[33,34,35]

SSEA (Stage-specific embryonic antigen-4); VEGF (vascular endothelial growth factor); HGF (hepatocyte growth factor); BDNF (brain-derived neurotrophic factor); IGF (insulin-like growth factor); bFGF (basic fibroblast growth factor); TGF (transforming growth factor); Ang (Angiotensin); EPO (Erythropoietin); SCF (Stem Cell Factor); PDGF (platelet-derived growth factor); PG-CSF (Granulocyte Colony-Stimulating Factor); GM-CSF (Granulocyte-Macrophage Colony-Stimulating Factor); EGF (epidermal growth factor); TNF (tumor necrosis factor); IFN (Interferon); and KGF (Keratinocyte Growth Factor).

## Data Availability

Not applicable.
